# Optimization of Antioxidant Potential of *Penicillium granulatum* Bainier by Statistical Approaches

**DOI:** 10.5402/2012/452024

**Published:** 2012-05-15

**Authors:** Priyanka Chandra, Daljit Singh Arora

**Affiliations:** Microbial Technology Laboratory, Department of Microbiology, Guru Nanak Dev University, Amritsar 143005, India

## Abstract

A three-step optimization strategy which includes one-factor-at-a-time classical method and different statistical approaches (Plackett-Burman design and response surface methodology) that were applied to optimize the antioxidant potential of *Penicillium granulatum.* Antioxidant activity was assayed by different procedures and compared with total phenolic content. Primarily, different carbon and nitrogen sources were screened by classical methods, which revealed sucrose and NaNO_3_ to be the most suitable. In second step, Plackett-Burman design also supported sucrose and NaNO_3_ to be the most significant. In third step, response surface analysis showed 4.5% sucrose, 0.1% NaNO_3_, and incubation temperature of 25°C to be the optimal conditions. Under these conditions, the antioxidant potential assayed through different procedures was 78.2%, 70.1%, and 78.9% scavenging effect for DPPH radical, ferrous ion, and nitric oxide ion, respectively. The reducing power showed an absorbance of 1.6 with 68.5% activity for FRAP assay.

## 1. Introduction

Antioxidants are the compounds that scavenge or neutralize free radicals. These are produced constantly in human body during normal physiological processes and are responsible for various pathological process like aging, cardiovascular diseases, diabetes, cancer, Alzheimer's disease, neurodegenerative disorders, atherosclerosis, cataracts, and inflammation [1].

Filamentous fungi are historically important sources of pharmacologically relevant secondary metabolites, and this group continues to provide new structures with novel biological activities [2]. The discovery of pharmaceutically significant novel product through screening of microbial secondary metabolites is an attraction in the pharmaceutical and biotechnological industry and is also becoming increasingly beneficial. There is widespread acceptance that fungi are virtually unlimited source of novel structures with many potential therapeutic applications including antibacterial, anticancer, anti-inflammatory, immunostimulatory, and antioxidant [3]. Recently, fungi have emerged as an important source of antioxidant compounds [4, 5]. Phenolic compounds are considered to be the main secondary metabolites in plants, mushrooms, and fungi responsible for their antioxidant activity [6–8].

Physiochemical and nutritional conditions greatly influence the growth as well as the activities of the microorganisms. Hence, optimization of such parameters is an important step for the enhancement of activity. Optimization is a tedious process due to involvement of multivariable parameters. The conventional one-factor-at-a-time approach of optimization is not only time consuming but often incapable of reaching the true optimum conditions. In the optimization process, screening of important factors is initially carried out, and these selected factors are then optimized by different techniques. The Plackett-Burman design is a well-known and widely used statistical technique for screening and selection of most significant culture variables. Response surface methodology (RSM) is a three-factorial design that gives relationship between one or more measured dependent responses with a number of input (independent) factors. RSM has some advantages that include less experiment numbers, suitability for multiple factor experiments, search for relativity between factors, and finding of the most suitable conditions and forecast response [9]. In this paper, linear or quadratic effects of experimental variables help to construct contour plots and a model equation fitting the experimental data. This facilitates the determination of optimum value of factors under investigation and prediction of response under optimized condition [10].

The present study thus aims to optimize the conditions for enhancement of antioxidant activity of soil fungal isolate *Penicillium granulatum* Bainier by various assay procedures [1,1-diphenyl-2-picryl hydrazyl (DPPH) assay, reducing power, ferrous ion and nitric oxide ion scavenging activity, ferric reducing antioxidant power (FRAP) assay] using one-factor-at-a-time classical approach, Plackett-Burman design, and response surface methodology. An effort has been made to work out the correlation (if any) between antioxidant activity and total phenolic content.

## 2. Materials and Methods

### 2.1. Experimental

*Penicillium granulatum* Bainier was isolated from soil of Amritsar, Punjab, India (31°37′59′′ North, 74°51′56′′ East) and identified on the basis of standard protocol and the identity was confirmed by National Fungal Culture Collection of India, Agharkar Research Institute, Pune, India. To study the antioxidant potential, the fungus was grown on 50 mL Czapek Dox's broth (sucrose 3%, NaNO_3_ 0.2%, K_2_HPO_4 _0.1%, MgSO_4 _0.05%, KCl 0.05%, FeSO_4_ 0.001%). The medium was inoculated with two discs (8 mm) of fungal mycelia obtained from 6 to 7 days grown culture on Yeast extract glucose agar plates. The growth was carried out under stationary conditions at 25°C. After incubation for 10 days, the culture broth was filtered through Whatmann filter paper no. 1, and the filtrate so obtained was analyzed for antioxidant potential by different assay procedures, and total phenolic content was estimated with Folin-Ciocalteau (FC) method.

### 2.2. Assay Procedures for Antioxidant Activity

Different assay procedures for antioxidant activity were used as described earlier [11]. The scavenging activity for DPPH free radicals was measured according to Zhao et al. [12] with slight modifications using 0.1 mM DPPH solution in ethanol, and the decolourization of DPPH was determined by measuring the decrease in absorbance at 517 nm, and the DPPH radical scavenging effect was calculated according to the following equation:

(1) $$\%\;\text{scavenging}\;\text{rate}\;=\;\left[1-\frac{\left(A1-A2\right)}{A0}\right]\times \;100,$$

where *A*0 represents the absorbance of the control (DPPH without extract), *A*1 represents the absorbance of the reaction mixture, and *A*2 represents the absorbance without DPPH (DPPH was replaced by same volume of distilled water).

The reducing power of the extracts was determined according to Chang et al. [13] using potassium ferricyanide, trichloroacetic acid, and FeCl_3_. Absorbance was read at 700 nm to determine the amount of ferric ferrocyanide (Prussian blue) formed. Higher absorbance of the reaction mixture indicates higher reducing power of the sample.

Ferric reducing antioxidant power (FRAP) assay was carried out according to Othman et al. [14] by monitoring the reduction of Fe^3+^-tripyridyl triazine (TPTZ) to blue colored Fe^2+^-TPTZ. The absorbance was measured at 593 nm. Antioxidant potential of the sample was compared with the activity of FeSO_4_.

Ferrous ion scavenging (metal chelating) activity of the extracts was measured according to Zhao et al. [12] using FeCl_2_ and ferrozine and the absorbance measured at 562 nm. The chelating activity was calculated as

(2) $$\text{Chelating}\;\text{rate}=\left[1-\frac{\left(A1-A2\right)}{A0}\right]\times 10,$$

where *A*0 represents the absorbance of the control (without extract) *A*1 represents the absorbance of reaction mixture, and *A*2 represents the absorbance without FeCl_2_.

Nitric oxide production from sodium nitroprusside was measured according to Ki et al. [15] using sodium nitroprusside solution and Griess reagent. The absorbance was taken at 546 nm and compared with absorbance of 1 mg/mL of standard solution (sodium nitrite) treated in the same way with Griess reagent.

### 2.3. Determination of Total Phenolic Content (TPC)

The total phenolic content was determined colorimetrically using the Folin-Ciocalteau (FC) method according to Singleton et al. [16] with some modifications [11], and the absorbance of the reaction mixture was measured at 765 nm. Gallic acid was taken as standard.

### 2.4. Determination of Mycelial Biomass

The dry weight of mycelia was measured after repeated washing of the fungal biomass with distilled water and drying overnight at 70°C to a constant weight [17].

### 2.5. Medium Optimization Using One-Factor-at-a-Time Classical Method

To find out the best carbon source, sucrose in the Czapek Dox's medium was replaced with same concentration of one of the sugars (glucose, maltose, lactose, and starch, and glycerol) and to work out the best nitrogen source, NaNO_3_ in Czapek Dox's medium was substituted with one or the other inorganic nitrogen source (KNO_3_, NH_4_NO_3_, (NH_4_)_2_Cl, (NH_4_)_2_ SO_4_, (NH_4_)H_2_ SO_4_) or nitrogen-rich organic supplement (yeast extract, peptone, malt extract, urea, casein, soyabean meal).

### 2.6. Statistical Optimization of the Medium

#### 2.6.1. Plackett-Burman Experimental Design

The Plackett-Burman experimental design is a valuable tool for the rapid evaluation of the effects of various medium components. Because this design is a preliminary optimization technique, which tests only two levels of each medium component, it cannot provide the optimal quantity of each component required in the medium. This technique, however, provides indications of how each component tends to affect the activity. The screening of most significant parameters affecting antioxidant potential was studied by the Plackett-Burman design. The 5 factors, which are components of Czapek Dox's medium (sucrose, NaNO_3_, K_2_HPO_4,_ KCl, and MgSO_4_), were examined. Total 14 tests were designed including 12 combinations and 2 repetitions at central point which contain different concentration of each factor, and the effect of each factor was determined by the difference between the average of the + and − responses. The significance level of effect of each factor was determined by student's *t* test.The most common mean of assessing significant value is the *P* value which was also evaluated for each factor.

#### 2.6.2. Response Surface Methodology through Box-Behnken Designs

On the basis of results from screening of different carbon and nitrogen sources through one-factor-at-a-time classical method and different components by Plackett-Burman design, sucrose and NaNO_3_ were found to be the best for antioxidant activity. Sucrose as carbon source, NaNO_3_ as nitrogen source and temperature were taken independent variables for the optimization by RSM using Box-Behnken designs of experiments. Each variable was studied at three levels (−1, 0, +1); for sucrose these were 5%, 3%, and 1%; NaNO_3_: 0.05%, 0.2%, and 0.35%; temperature: 15°C, 25°C, and 35°C.

The experimental design included 17 flasks with five replicates having all the three variables at their central coded values. The DPPH assay, reducing power, ferrous ion, and nitric oxide ion scavenging activity, FRAP assay, and their total phenolic contents were taken as responses *G*_(1–6)_. The mathematical relationship of response *G* (for each parameter) and independent variable *X* (*X*_1_, sucrose; *X*_2_, NaNO_3_; *X*_3_, temperature) was calculated by the following quadratic model equation:

(3) $$\begin{array}{cc} G_{\left(1-6\right)} & =\;\beta_{0}+\beta_{1}X_{1}\;+\beta_{2}X_{2}\;+\beta_{3}X_{3}\;\\ & \;+\beta_{11}{X^{2}}_{1}+\beta_{22}{X^{2}}_{2}\;+\;\beta_{33}{X^{2}}_{3}\;\\ & \;+\beta_{12}X_{1}X_{2}+\beta_{13}X_{1}X_{3}+\beta_{23}X_{2}X_{3}, \end{array}$$

where *G* is the predicted response; *β*_0_, intercept; *β*_1_, *β*_2_, and *β*_3_, linear coefficients; *β*_11_, *β*_22_ and *β*_33_, squared coefficients *β*_12_, *β*_13_, and *β*_23_ interaction coefficients. MINITAB version 11 statistical software was used to obtain optimal working conditions and generate response surface graphs. Statistical analysis of experimental data was also performed using this software.

### 2.7. Toxicity Tests

The culture broth used to assess the antioxidant activity was subject to Ames test by using *Salmonella* reverse mutation based on histidine dependence and mutations in *S*.* typhimurium *[18]. Cytotoxicity was tested by using 3-(4,5-dimethylthiazol-2-yl)-2,5-diphenyl tetrazolium bromide (MTT) method. The fungal extracts (100 *μ*l) were incubated with 1 × 10^5 ^RBCs/well in 96-well ELISA plates for 24 h. Then 100 *μ*l MTT solution (0.5%, w/v) was added to each well and incubated further for 4 h. After incubation, the supernatant was removed, and 100 *μ*l DMSO was added to each well to dissolve the formazan crystals. The absorbance was measured at 590 nm using an automated microplate reader. The wells with untreated cells served as control [19].

## 3. Results and Discussion

Fungi are recognized as prolific secondary metabolite producers and have provided several bioactive compounds and chemical models, currently used as pharmaceuticals. Recently, they have attracted the attention of scientific community to produce wide range of secondary metabolites possessing antioxidant activity along with their amenability to easy manipulations. Nutritional and fermentation conditions have a great influence on secondary metabolites production. Optimization of fermentation medium and conditions is very important for maximizing the yield and minimizing the production cost of many secondary metabolites. Most of the recent optimization efforts have relied on statistical experimental design and response surface analyses [20]. Thus, the present study is based on statistical design as it is a powerful tool that can be used to account for the main as well as interactive influences of fermentation parameters on the process performance. It is an efficient way to generate useful information with limited experimentation, thereby cutting the process development time and cost [21].

Effects of different carbon and nitrogen sources on antioxidant potential were studied by various assay procedures. Initially, to assess the antioxidant potential by various assay procedures all the experimentation was done by growing *Penicillium granulatum* on Czapek Dox's broth medium. Carbohydrates are the structural and storage compounds in the cells of fungi and thereby play a key role in the growth as well as in the production of various useful secondary metabolites. Of the various carbon sources tested, sucrose supported the maximum antioxidant activity by* Penicillium granulatum *which is in consonance with earlier studies carried out on *Aspergillus candidus* [22]. Though, it contravenes the general perception that glucose and starch are known to be the best carbon source for the fungal growth. The order for different carbon sources supportive of best antioxidant activity was found to be sucrose > dextrose > maltose > lactose > starch > glycerol (Table 1). Sucrose was thus selected as the carbon source for further experimentation, which was also found to be the best during earlier studies on *Aspergillus* spp. [11].

Similarly, NaNO_3_ turned out to be the best nitrogen source to support maximum antioxidant potential. Peptone and yeast extract were other nitrogen-rich sources while urea gave the poorest activity. NO ion scavenging activity was monitored for 180 min (data not shown) which increased gradually with respect to time, however, data recorded at 180 min is only shown (Table 2). The antioxidant profile of *Penicillium granulatum* for different carbon and nitrogen sources remained the same irrespective of assay procedures adopted, which also shows the ability of extract to scavenge different types of free radicals.

The above results thus explain that a fungal species may have the ability to utilize a particular carbon source for vegetative growth but may not be able to use it for production of specialized structural molecules. Sucrose and NaNO_3_ are regarded as sustainable sources, which favors the production of secondary metabolites [23]. The study thus demonstrated the basic composition of Czapek Dox's medium to be the best for effective antioxidant activity in consonance with earlier studies [11]. In fact, the designing of culture media has a major impact on the growth of microbes and the production of microbial products [24].

Biomass did not significantly affect the activity, as dextrose-supported maximum biomass followed by starch but maximum antioxidant potential, as assayed by different procedures, was demonstrated by sucrose. Similarly, yeast extract followed by peptone supported maximum biomass but sodium nitrate showed highest activity. However, urea gave minimum biomass accompanied by minimum activity. These observations are in agreement with earlier study on *Lentinula edodes *which also did not show any correlation between mycelial biomass and antibacterial activity [25].

The total phenolic content (TPC) of *Penicillium granulatum* extracts has been expressed as gallic acid equivalent (GAE), that is, mg gallic acid/100 mL culture. TPC is known to be responsible for antioxidant activity, and the *Penicillium granulatum* possessed high TPC, which is positively correlated with its antioxidant potential. The highest TPC yield was 7.28 mg/mL in the presence of sucrose and NaNO_3_ in the medium. On the basis of above results, Czapek Dox's broth medium was chosen for remaining experimentations. It is commonly known that the antioxidative effects are mainly due to redox properties of phenolic compounds which can play an important role in absorbing and neutralizing free radicals by acting as reducing agents and hydrogen donor or quenching singlet and triplet oxygen or decomposing peroxides [4]. The importance of phenolic contents has been endorsed by their high content in *Penicillium granulatum,* and their antioxidant activity is quite comparable to that of many mushrooms as well as medicinal plants.

### 3.1. Plackett-Burman Design for Selection of Significant Components

A Plackett-Burman design experiment was employed to evaluate the influence of five factors (sucrose, NaNO_3_, K_2_HPO_4,_ KCl, and MgSO_4_) and their importance in culture medium to obtain better antioxidant activity. Antioxidant potential of *Penicillium granulatum* assayed by different procedures and extracellularly produced total phenolic content varied significantly with the 14 run of different combinations of the media components (Table 3). The maximum antioxidant potential along with high TPC was observed in run order 13 and run order 14 which was followed by run order 5. The results were subjected to regression analysis and the analysis of variance (ANOVA) which revealed sucrose and NaNO_3_ to have statistically significant effect on antioxidant potential with *P* value ≤0.05 and ≤0.5, respectively which showed that of the five variables only sucrose and NaNO_3_ played a critical role for antioxidant activity. Based on these results, sucrose and NaNO_3_ were selected as two variables and applied to optimize the medium composition by RSM. To know the optimum temperature and its interaction with other variables (sucrose and NaNO_3_), it was chosen as a third variable as it is the important physical parameter that affects the activity as well as fungal growth.

### 3.2. Box-Behnken Design

#### 3.2.1. Fitting the Model

The data obtained from quadratic model equation was found to be significant. It was verified by *F* value and the analysis of variance (ANOVA) by fitting the data of all independent observations in response surface quadratic model (Table 4). *R* ^2^ value for all the responses ranged between 86 and 95% which showed suitable fitting of the model in the designed experiments (Table 5). The final predictive equations for each response: DPPH assay (*G*_1_), reducing power (*G*_2_), ferrous ion scavenging activity (*G*_3_), FRAP assay (*G*_4_) and nitric oxide ion scavenging activity (*G*_5_), and their total phenolic contents (*G*_6_) obtained are as follow:

(4) $$\begin{array}{cc} G_{\left(1\right)} & =-13.71+24.75X_{1}+142.02X_{2}\;\\ & \;+2.00X_{3}-2.74{X^{2}}_{1}+19.62{X^{2}}_{2}\\ & \;-0.03{X^{2}}_{3}-47.50X_{1}X_{2}\\ & \;+\;0.07X_{1}X_{3}-0.49X_{2}X_{3},\\ G_{\left(2\right)} & =-1.483+\;0.504X_{1}+3.966X_{2}\\ & \;+0.080X_{3}-0.050{X^{2}}_{1}+0.989{X^{2}}_{2}\\ & \;-\text{–}0.001{X^{2}}_{3}-1.450X_{1}X_{2}\\ & \;+0.005X_{1}X_{3}-0.010X_{2}X_{3},\\ G_{\left(3\right)} & =-7.73\;+\;21.60X_{1}+104.91X_{2}\\ & \;+1.75X_{3}-\text{–}2.45{X^{2}}_{1}+46.89{X^{2}}_{2}\\ & \;-0.02{X^{2}}_{3}-38.58X_{1}X_{2}+0.04X_{1}X_{3}\\ & \;-0.60X_{2}X_{3},\\ G_{\left(4\right)} & =-1.38+18.06X_{1}+101.89X_{2}\\ & \;+1.51X_{3}-1.92{X^{2}}_{1}-8.89{X^{2}}_{2}-0.02{X^{2}}_{3}\\ & \;-38.17X_{1}X_{2}+0.05X_{1}X_{3}+0.15X_{2}X_{3},\\ G_{\left(5\right)} & =-1.57+\;21.30X_{1}+114.35X_{2}\\ & \;+1.48X_{3}-2.57{X^{2}}_{1}+110.38{X^{2}}_{2}\\ & \;-0.02{X^{2}}_{3}-40.92X_{1}X_{2}+0.11X_{1}X_{3}\\ & \;-1.67X_{2}X_{3},\\ G_{\left(6\right)} & =-21.30+6.87X_{1}+40.84X_{2}\;\\ & \;+1.00X_{3}-0.64{X^{2}}_{1}+21.22{X^{2}}_{2}\\ & \;-0.01{X^{2}}_{3}-11.50X_{1}X_{2}\\ & \;-0.02X_{1}X_{3}-0.92X_{2}X_{3}. \end{array}$$

The optimized values of factors were validated by repeating the experiment in triplicate flasks. 

#### 3.2.2. Effect of Different Variables on DPPH Assay

**Figure 1:**
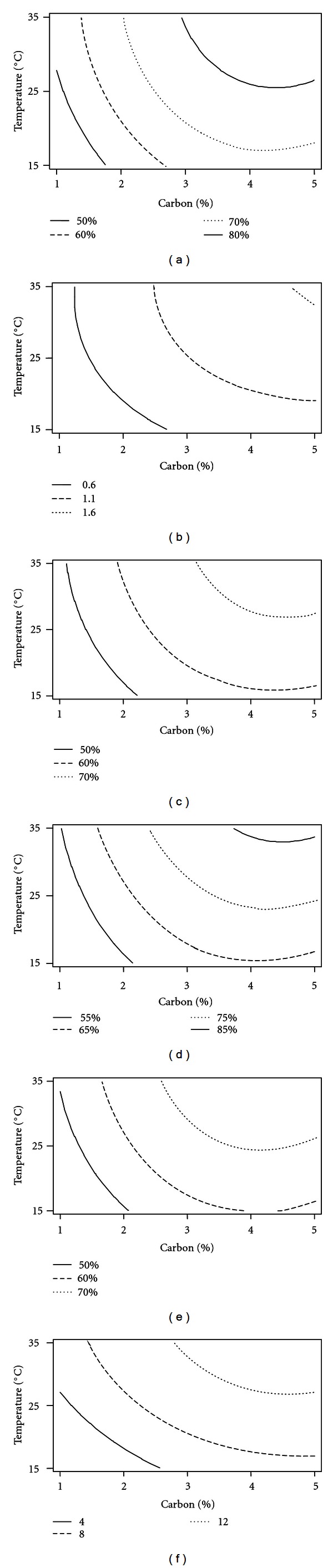
(a–f) Contour plot showing effect of different variables on antioxidant potential (hold value: 0.05% of sodium nitrate). (a) DPPH assay (% activity); (b) reducing power (absorbance); (c) FRAP assay (% activity); (d) nitric oxide ion scavenging activity (% activity); (e) ferrous ion scavenging activity (% activity); (f) total phenolic content (mg/ml).

Sucrose significantly affected the DPPH activity. The linear effect (*X*_1_), squared effect (*X*_1_^2^) as well as interactive effect (*X*_1_ *X*_2_) were highly significant (*P* value < 0.005). Linear effect (*X*_2_) was significant with *P* value <0.5. The response surface graphs showed the highest activity at concentration of sucrose between 3 and 5% but with least amount of NaNO_3_. Maximum predicted value for DPPH scavenging effect (80%) was obtained at 4.5% of sucrose, 0.05% of NaNO_3_ and at 25°C (Figure 1(a)).

#### 3.2.3. Effect of Variables on Reducing Power

Linear effects (*X*_1_, *X*_2_, *X*_3_) and squared effects (*X*_1_^2^) were significant with *P* value <0.5. Interactive effect (*X*_1_ *X*_2_) was most significant at *P* value ≤ 0.005. The response surface graphs showed the highest reducing potential with an absorbance of 1.6, when the concentration of sucrose is 4.5% with 0.05% of NaNO_3_ and at a temperature of 35°C (Figure 1(b)). 

#### 3.2.4. Effect of Variables on FRAP Assay, Ferrous Ion, and Nitric Oxide Ion Scavenging Activity

For FRAP assay, interactive effect (*X*_1_ *X*_2_), linear (*X*_1_), and its squared effect (*X*_3_^2^) showed significance at *P* ≤ 0.05. Interactive effect (*X*_1_ *X*_2_) was significant with *P* value ≤ 0.005, while linear effect (*X*_1_, *X*_2_) showed significance at *P* ≤ 0.05 for ferrous ion scavenging activity. Linear effect (*X*_1_, *X*_2_) was significant with *P* value ≤ 0.005 and interactive effect (*X*_1_ *X*_2_) showed significance at *P* ≤ 0.05 for nitric oxide ion scavenging activity. Ferric reducing antioxidant power was maximum (70%) at 30°C, with medium composition of 4% of sucrose with 0.05% of NaNO_3_ (Figure 1(c)). Similarly, highest scavenging effect of 85% for nitric oxide ion was observed at 35°C with 4.5% and 0.05% of sucrose and NaNO_3 _  respectively (Figure 1(d)). The chelating effect was highest (70%) at 25°C in the medium containing 4% of sucrose with 0.05% of NaNO_3 _ (Figure 1(e)). Antioxidant potential as assayed by different procedures demonstrated decrease in activity with the further increase of NaNO_3 _  concentration and decrease in the temperature and sucrose concentration.

#### 3.2.5. Effect of Variables on Total Phenolic Content

The interactive effect (*X*_1_ *X*_2_) was highly significant with *P* value ≤ 0.005 while linear (*X*_1_) and squared effect of sucrose (*X*_1_^2^) and interactive between sucrose-temperature (*X*_1_ *X*_3_) is significant with *P* value ≤ 0.05. The highest amount of TPC was obtained at 4% of sucrose and with 0.05% of NaNO_3_ concentration at 25°C (Figure 1(f)) and yield decreased with the decrease in temperature and sucrose concentration and with the increase in NaNO_3_ concentration.

#### 3.2.6. Validation of Results

On the basis of the results, sucrose and NaNO_3_ were found to be significant, thus, endorsing the important role played by carbon and nitrogen source to regulate secondary metabolite production and the microbial growth [26].

Though KCl, MgSO_4 _  and K_2_HPO_4_ did not significantly affect the antioxidant activity but are retained at standard concentration in Czapek Dox's medium because magnesium and potassium are required by all the fungi for a variety of regulatory functions and control the biosynthesis of various secondary metabolites. This shows that the medium most suitable for growth may or may not be equally effective for secondary metabolites, and thus enhancement of secondary metabolites can only be achieved through systematic manipulation of different parameters [27].

Thus, from the overall assessment, the optimized conditions for different assay procedures may be concluded as, 4.5% sucrose, 0.05% NaNO_3_, and incubation temperature of 25°C while other media components were retained as standard concentration in Czapek Dox's medium. The *F* value and *R* ^2^ value showed that the model correlated well with measured data and was statistically significant. To confirm the model adequacy for predicting maximum scavenging activity, the verification experiment using the optimum medium composition (mentioned above) was performed. The experiments under optimized conditions were carried out in triplicates which showed 78.2%, 70.1% and 78.9% scavenging effect for DPPH radical, ferrous ion, and nitric oxide ion, respectively. The yield for TPC was 12.3 mg/mL, and reducing potential showed an absorbance of 1.6 with 68.5% activity for FRAP assay. The results showed the scavenging effect for DPPH radical, ferrous ion and nitric oxide ion was enhanced by 1.0, 1.0, and 1.15 folds, respectively while reducing potential and ferric reduction rate was enhanced by 1.7 and 1.1 folds. The production of TPC was enhanced by 1.7 folds. A good agreement between the predicted and experimental results verified the validity of the model and the improvement of antioxidant activity indicated that RSM is a powerful tool for determining the exact optimal values of the individual factors and the maximum response value.

### 3.3. Toxicity Tests

The cell-free fungal extracts, when studied for Ames test, showed no mutagenic activity as no bacterial colony was observed on agar plates containing fungal extracts, while more that 1000 colonies were observed on positive control (sodium azide) containing plate. Similarly, results obtained from MTT assay revealed that the cell-free extracts were noncytotoxic and showed much higher absorbance (0.762) as compared to positive control (0.101).

### 3.4. Correlation between Antioxidant Activity and TPC

The results obtained indicate *Penicillium granulatum* to be a potent antioxidant producer having broad spectrum against various free radicals. To measure the total antioxidant potential using a single assay procedure seems to be unrealistic, yet there are numerous published methods claiming to measure total antioxidant activity *in vitro* [28]. Thus, to validate the reliability of different methods, the correlation between the different assay procedures is necessary. Previous studies have shown the linear correlation between total phenolic content and antioxidant activity [13] in this study too, total phenolic content of *Penicillium granulatum* correlated well with the antioxidant activity. The better production of phenolics under optimized conditions also resulted in enhanced antioxidant activity. The extract obtained from *Penicillium granulatum* showed good activity against DPPH radical by neutralizing the free radical character of purple color DPPH, either by transfer of electron or hydrogen atom, to yellow-colored diamagnetic molecule revealing hydrogen donating property of phenolic compounds present in the extract which can be supported by the positive correlation (*r* = 0.835) between the results of DPPH assay and TPC [29]. Similarly, positive correlation (*r* = 0.852) was found between reducing power assay and TPC. Reducing power assay proves the potential of the phenolic compounds in the extracts to act as reductones that inhibit lipid peroxidation by donating a hydrogen atom thereby terminating the free radical chain reaction. Moreover, this reducing potential may be due to the di or monohydroxy substitution in the aromatic rings that possess potent hydrogen-donating ability [12]. Results of FRAP assay are also positively correlated (*r* = 0.845) with TPC, and good activity of the fungal extract for FRAP assay denotes its reducing potential. Generally the reducing properties are associated to the breaking of free radical chain by donating a hydrogen atom [14]. The extracts also showed appreciable chelating activity of metals, as the transition metals such as ferrous ion can stimulate lipid peroxidation by generating hydroxyl radicals through Fenton reaction. The chelating activity for ferrous ion was assayed by the inhibition of formation of red-colored ferrozine and ferrous complex. There was positive correlation (*r* = 0.830) between chelating activity and TPC [12]. As evident from studies, the extracts are able to scavenge nitric oxide ion, and correlation between TPC was found to be positive (*r* = 0.826). NO^•^ is an effective reactive radical that acts as an important oxidative biological signaling molecule in a large variety of diverse physiological processes, including neurotransmission, blood pressure regulation, defense mechanisms, smooth muscle relaxation and immune regulation. Overproduction of reactive nitrogen species is called nitrosative stress. Nitrosative stress may lead to nitrosylation reactions that can alter the structure of proteins and so inhibit their normal function and act as a potent-oxidizing agent that can cause DNA fragmentation and lipid peroxidation [30]. 

Most of the literature available is on antioxidant activity of plants and mushrooms though some of the fungi are known to produce antioxidant activity. To the best of our knowledge apparently this is the first systematic report on antioxidant activity of *Penicillium granulatum* demonstrated by different assay procedures and its optimization by statistical methods. These results are comparable with the antioxidant activity of various other fungi, *Aspergillus candidus*, *Chaetomium* sp.,* Cladosporium* sp.,* Colletotrichum gloeosporioides* [31] and many mushrooms such as *Lentinus edodes*, *Volvariella volvacea* [32] and many medicinal plants like *Amaranthus paniculatus, Aerva lanata, Coccinia indica*, and *Coriandrum sativum *[33].

## 4. Conclusions

The study thus suggests that not only mushrooms and plants but some other fungi may also be a good source of antioxidant compounds, and *Penicillium granulatum* is one such potential candidate offering a better scope for production and easier downstreaming of such bioactive compounds. These findings will facilitate the further studies to gain better understanding of production of bioactive metabolites in fungi, which will be helpful in their biotechnological mass production in near future.

## Figures and Tables

**Table 1:** Effect of various carbon sources on antioxidant potential of *Penicillium granulatum. *

% activity	Dextrose	Maltose	Lactose	Starch	Glycerol	Sucrose
DPPH^a^ assay	72 ± 0.2	62.8 ± 0.11	64.3 ± 0.1	60.2 ± 0.1	50.2 ± 0.9	72.4 ± 0.21
Reducing power	0.90 ± 0.8	0.53 ± 0.8	0.686 ± 0.2	0.52 ± 0.02	0.32 ± 0.08	0.980 ± 0.18
Fe^2+^ scavenging activity	64.8 ± 0.3	54.3 ± 0.2	45.2 ± 0.32	40.3 ± 0.2	42.4 ± 0.5	64.2 ± 0.17
FRAP^b^ assay	60.2 ± 0.21	50.8 ± 0.3	40.3 ± 0.35	38.2 ± 0.5	40.2 ± 0.2	61.9 ± 0.04
NO^c^ scavenging activity						
30 min	46.2 ± 0.03	20.8 ± 0.01	20.1 ± 0.2	22.1 ± 0.02	22.7 ± 0.2	35.01 ± 0.01
60 min	48.0 ± 0.1	37.3 ± 0.1	24.2 ± 0.1	24.2 ± 0.31	25.1 ± 0.05	42.8 ± 0.01
90 min	51.4 ± 0.9	41.2 ± 0.5	36.7 ± 0.011	29.3 ± 0.011	28.9 ± 0.1	53.16 ± 0.16
120 min	60.3 ± 0.1	48.2 ± 0.06	38.4 ± 0.01	37.4 ± 0.71	30.5 ± 0.2	62.98 ± 0.04
180 min	65.2 ± 0.1	52.3 ± 0.5	42.3 ± 0.9	40.7 ± 0.8	38.5 ± 0.05	68.2 ± 0.21
TPC^d^ (mg/mL)	7.1 ± 0.03	4.0 ± 0.1	4.3 ± 0.02	3.4 ± 0.02	2.2 ± 0.06	7.28 ± 0.02
Biomass (g/L)	4.5 ± 0.005	2.3 ± 0.045	2.01 ± 0.076	4.08 ± 0.09	1.50 ± 0.02	3.05 ± 0.005

^a^DPPH-1.1-diphenyl-2-picryl hydrazyl; ^b^FRAP- ferric reducing antioxidant power; ^c^NO-nitric oxide; ^d^TPC-total phenolic content.

**Table 2:** Effect of various nitrogen sources on antioxidant potential of *Penicillium granulatum. *

Nitrogen sources	DPPH^a^ assay	Reducing power	Fe^2+^ scavenging activity	FRAP^b^ assay	NO^c^ scavenging activity	TPC^d^ (mg/mL)	Biomass (g/L)
Nitrogen rich organic supplements							
Yeast extract	72.0 ± 0.12	0.9 ± 0.8	63.2 ± 0.02	60.2 ± 0.1	65.8 ± 0.21	7.3 ± 0.33	4.2 ± 0.08
Peptone	71.2 ± 0.3	0.93 ± 0.6	60.8 ± 0.52	59.8 ± 0.2	63.2 ± 0.5	6.9 ± 0.1	4.25 ± 0.05
Malt extract	62.6 ± 0.1	0.62 ± 0.02	50.2 ± 0.2	48.2 ± 0.6	45.4 ± 0.91	4.0 ± 0.12	3.0 ± 0.08
Casein	60.8 ± 0.2	0.374 ± 0.23	32.7 ± 0.3	30.2 ± 0.3	27.3 ± 0.1	3.1 ± 0.42	3.0 ± 0.002
Soyabean meal	68.2 ± 0.56	0.63 ± 0.1	60.2 ± 0.56	55.8 ± 0.67	56.3 ± 0.54	4.1 ± 0.21	2.45 ± 0.08
Urea	30.8 ± 0.4	0.103 ± 0.21	22.3 ± 0.34	20.8 ± 0.78	—	1.2 ± 0.34	0.9 ± 0.06
Inorganic nitrogen sources							
KNO_3_	60.3 ± 0.3	0.50 ± 0.45	56.3 ± 0.56	54.2 ± 0.98	50.2 ± 0.32	3.4 ± 0.21	2.3 ± 0.07
(NH_4_)_2_ SO_4 _	53.8 ± 0.56	0.29 ± 0.6	50.6 ± 0.78	49.7 ± 0.21	48.8 ± 0.5	2.8 ± 0.03	2.25 ± 0.07
(NH_4_)H_2_ SO_4_	50.6 ± 0.2	0.22 ± 0.8	42.8 ± 0.89	40.6 ± 0.3	36.7 ± 0.8	2.1 ± 0.04	2.3 ± 0.03
NH_4_NO_3_	55.4 ± 0.6	0.31 ± 0.98	53.2 ± 0.34	51.2 ± 0.9	50.2 ± 0.5	3.1 ± 0.7	2.3 ± 0.06
NaNO_3_	72.4 ± 0.21	0.98 ± 0.18	64.2 ± 0.17	61.9 ± 0.04	68.2 ± 0.21	7.28 ± 0.02	3.0 ± 0.005
(NH_4_)_2_Cl	60.2 ± 0.2	0.47 ± 0.24	54.8 ± 0.23	52.8 ± 0.5	50.6 ± 0.4	3.2 ± 0.5	2.45 ± 0.07

^a^DPPH-1.1-diphenyl-2-picryl hydrazyl; ^b^FRAP- ferric reducing antioxidant power; ^c^NO-nitric oxide; ^d^TPC-total phenolic content.

**Table 3:** Plackett-Burman design variables with different antioxidant potential as response.

	Variables (%)					Antioxidant activity (% activity)					
Run	Sucrose	NaNO_3_	K_2_HPO_4_	MgSO_4_	KCl	DPPH Assay	Reducing power	Fe^2+^ scavenging activity	FRAP assay	NO scavenging activity	TPC (mg/mL)
1	5.0	0.000	0.18	0.000	0.000	60.8	0.63	50.8	46.7	52.7	4.1
2	5.0	0.350	0.00	0.090	0.000	70.5	0.73	60.9	58.6	63.8	5.1
3	0.0	0.350	0.18	0.000	0.090	22.5	0.22	15.5	13.2	16.7	1.2
4	5.0	0.000	0.18	0.090	0.000	50.7	0.58	40.9	40.8	45.3	3.2
5	5.0	0.350	0.00	0.090	0.090	72.8	0.96	63.8	60.5	66.8	6.2
6	5.0	0.350	0.18	0.000	0.090	64.5	0.68	55.7	52.8	60.9	5.8
7	0.0	0.350	0.18	0.090	0.000	18.6	0.18	10.9	8.9	12.4	0.8
8	0.0	0.000	0.18	0.090	0.090	44.8	0.50	36.7	36.5	40.7	2.8
9	0.0	0.000	0.00	0.090	0.090	20.7	0.18	15.5	12.2	14.6	0.9
10	5.0	0.000	0.00	0.000	0.090	61.8	0.65	50.6	48.6	55.8	4.8
11	0.0	0.350	0.00	0.000	0.000	54.3	0.59	44.1	42.7	48.6	3.2
12	0.0	0.000	0.00	0.000	0.000	0.0	0.00	0.0	0.0	0.0	0.0
13	2.5	0.175	0.09	0.045	0.045	73.2	0.98	65.7	62.9	69.3	7.9
14	2.5	0.175	0.09	0.045	0.045	72.8	0.98	64.6	62.8	68.7	7.8

**Table 4:** Box-Behnken designs of different variables with their responses.

Variables (%)				Antioxidant activity (% activity)					
Run	Sucrose	NaNO_3_	Temperature	DPPH assay	Reducing power	Fe^2+^ scavenging activity	FRAP assay	NO scavenging activity	TPC (mg/mL)
1	1	0.05	25	50.20	0.540	48.3	46.1	52.90	3.1
2	5	0.05	25	77.40	1.320	68.9	66.9	74.80	12.6
3	1	0.35	25	76.20	1.260	66.6	65.2	72.90	6.4
4	5	0.35	25	46.40	0.300	40.9	40.2	45.70	2.1
5	1	0.2	15	45.50	0.345	40.7	41.9	44.80	2.2
6	5	0.2	15	48.10	0.460	42.3	43.2	47.60	2.8
7	1	0.2	35	68.30	0.770	62.3	60.4	62.80	6.9
8	5	0.2	35	76.20	1.250	66.8	65.8	74.80	6.1
9	3	0.05	15	66.90	0.650	60.5	58.6	64.60	4.2
10	3	0.35	15	67.90	0.689	62.9	54.5	70.60	5.7
11	3	0.05	35	75.40	1.200	66.7	65.7	74.90	12.1
12	3	0.35	35	73.44	1.180	65.5	62.5	70.90	8.1
13	3	0.2	25	72.70	0.980	64.2	61.8	68.90	7.4
14	3	0.2	25	73.80	1.020	64.3	61.8	68.90	7.8
15	3	0.2	25	72.80	0.970	64.7	61.6	68.90	8.5
16	3	0.2	25	73.90	1.100	65.7	62.6	69.99	8.6
17	3	0.2	25	72.06	1.100	65.8	64.7	70.10	8.3

**Table 5:** Regression coefficients for different antioxidant potential as responses.

Term	DPPH Assay	Reducing power	Fe^2+^ scavenging activity	FRAP assay	NO scavenging activity	TPC
Constant	−13.71	−1.483**	−7.73*	−1.38	−1.57	−21.30***
Sucrose	24.75***	0.504**	21.60**	18.06***	21.30**	6.87***
NaNO_3_	142.02*	3.966**	104.91*	101.89*	114.35*	40.84**
Temperature	2.00*	0.080**	1.75*	1.51*	1.48*	1.00**
Sucrose × sucrose	−2.74***	−0.050**	−2.45**	−1.92***	−2.57	−0.64***
NaNO_3_ × NaNO_3_	19.62	0.989	46.89	−8.89	110.38	21.22*
Temperature × temperature	−0.03*	−0.001	−0.02*	−0.02*	−0.02	−0.01*
Sucrose × NaNO_3_	−47.50***	−1.450***	−38.58***	−38.17***	−40.92***	−11.50***
Sucrose × temperature	0.07	0.005*	0.04	0.05*	0.11*	−0.02
NaNO_3_ × temperature	−0.49	−0.010	−0.60	0.15	−1.67*	−0.92**
*R* ^2^	90.7	94.1	86.8	92.1	87.6	94.8

*P ≤ 0.5; **P ≤ 0.05; ***P ≤ 0.005.

## Abbreviations

ANOVA: Analysis of variance
DPPH: 1,1-diphenyl-2-picryl hydrazyl
FC: Folin-Ciocalteau
FRAP: ferric reducing antioxidant power
GAE: Gallic acid equivalent
RSM: Response surface methodology
TPC: Total phenolic content.
